# Abstracts/Sommaires/Zusammenfassungen

**DOI:** 10.1155/S1463924679000535

**Published:** 1979


### Abstracts Sommaires Zusammenfassungen

### Page 189

**An Automatic Solution Making Device for Quality Control of Dyestuffs. Design Philosophy and Implementation.**
Hans-Christian Mez and Gero Michel, etc.

The paper describes an automatic system for the complete colorimetric assessment of dyestuffs. Evaluation of spectrophotometric transmission measurements of dyestuff solutions replaces the dyeing of textile samples and their visual comparison with standards. A mini computer controls the preparation of accurate solutions by a fully automated device. The hardware of this machine and the computer and software are the main subject of this paper.

**Un mécanisme automatique pour la fabrication de solutions pour le contrôle de qualité des teintures: la philosphie du dessein et la mise en oeuvre.**

Nous allons décrire un système automatique pour la quantification colorimetrique complète des colorants. L'évaluation des mesures spectrophotométriques de transmission remplace la teinture d'échantillons textils et leur comparaison visuelle avec des échantillons étalons. Un miniordinateur dirige la préparation des solutions à concentration connue à l'aide d'un appareil complètement automatisé. La construction de cet appareil, le miniordinateur et les programmes font l'objet de cet article.

**Ein automatisches Gerät für die Herstellung von Lösungen für die Qualitätskontrolle der Farbestoffe: die Philosophie des Entwurfes und die Erfüllung.**

Ein automatisches System zur vollständigen colorimetrischen Bewertung von Farbstoffen wird beschrieben. Die Auswertung spektrophotometrischer Transmissionsmessungen ersetzt das Färben von Textilmustern und den visuellen Vergleich derselben mit Eichfärbungen. Ein Minicomputer steuert die Herstellung von Losungen genau beakannter Konzentration mittels eines voll automatisierten Geräts. Der Aufbau dieses Apparats, der Minicomputer und die dazugehörige Software sind Gegenstand dieser Veroffentlichung.

### Page 195

**The measurement of splashover and carryover in centrifugal analyzers.** Peter Henry.

Splashover is an effect peculiar to centrifugal analyzers and is due to the bubble mixing process used to ensure complete mixing at the start of an analysis. A method is described whereby both splashover and the carryover due to the pipettor may be calculated from the results of the same analysis runs. It is shown that carryover is independant of splashover, but that more precise values of carryover are obtained when the values of splashover are low.

**Détermination des effets d'éclaboussure et de contamination dans les analysateurs à centrifugeuse**

Les effets d'éclaboussure sont propres aux analysateurs à centrifugeuse et proviennent du système de mélange à bulle, qui est nécessaire pour un mélange complet au début de l'analyse. Une méthode est décrite, qui permet de calculer l'effet de l'éclaboussure et de la contamination à partir des résultats de la même analyse. Il est démontré que la contamination est indépendante de l'éclaboussure mais que des valeurs plus précise sont obtenues quand la valeur de l'éclaboussure est basse.

**Die Bestimmung von Verschleppungs- und Ueberschwapp-Effekten in Zentrifugalanalysatoren.**

Ueberschwappeffekte sind Eigenheiten von Zentrifugalanalysatoren und stammen vom Gasblasen-Mischprozess, der zur Sicherstellung eines vollständigen Mischens zu Beginn der Analyse durchgeführt wird. Eine Methode wird beschrieben, mit der Ueberschwappeffekte und Verschleppung infolge der Pipettierung aus den Resultaten des gleichen Analysenlaufs berechnet werden können. Es kann gezeigt werden, dass die Verschleppung unabhängig ist von den Ueberschwappeffekten, dass aber umso genauere Werte für die Verschleppung erhalten werden, je kleiner die Werte für die Ueberschwappeffekte sind.

### Page 198

**A microprocessor controlled liquid chromatograph/atomic absorption sampling system.** Thomas M. Vickrey and William Eue.

A microprocessor controlled interface between a liquid chromatograph (LC) and a graphite furnace atomic absorption (AA) spectrometer is described. The LCAA interface utilizes a Motorola 6800 based Heathkit microprocessor trainer kit. The configuration described operates in the 'pulsed' sampling mode, and is capable of an injection of a coanalyte into the graphite furnace to remove chemical interferences. The effluent sampling precision and the precision of the coanalyte injection are described.

**Un système d'échantillonnage contrôlé par microprocesseur pour le couplage de la chromatographie en phase liquide avec la spectrometrie par absorption atomique**

Un instrument contrôlé par microprocésseur qui permet le couplage d'un chromatographe en phase liquide (LC) et d'un spectromètre pour absorption atomique (AA) avec four de graphite est décrit. Le coupleur LCAA est basé sur une boite de construction pour microprocesseur Heathkit avec le processeur Motorola 6800. La configuration décrite travaille avec échantillonnage pulsé et permet d'injecter un coanalyte afin d'eliminer des dérangements d'ordre chimique. La précision de l'échantillonnage a la sortie du LC et de l'injection du coanalyte sont décrites.

**Ein Probenahmesystem mit Mikroprozessorsteuerung für die Kombination der Flüssigchromatographie mit der Atomabsorption-Spektrometrie**

Es wird ein Zwischengerät mit Mikroprozessorsteuerung beschrieben, das die Kopplung eines Flüssigchromatographen (LC) mit einem Atomabsorptions-Spektrometer (AA) mit Graphitofen ermöglicht. Das LCAA Zwischengerät ist auf einem Heathkit Mikroprozessor Einführungs-Baukasten mit dem Motorola 6800 Prozessor aufgebaut. Die beschriebene Konfiguration arbeitet mit einer gepulsten Probenahme und ist befähigt, einen Koanalyten in den Graphitofen einzuspritzen um chemische Störungen zu beseitigen. Die Genauigkeit der Probenahme im Ausfluss und der Einspritzung des Koanalyten werden beschreiben.

### Page 202

**Assessment of the ENI Gemeni microprocessor-controlled centrifugal analyser**
N. Potezney, R.G. White and T.D. Geary.

The Electronucleonics Inc Gemeni is a low-priced microprocessor-controlled centrifugal analyser. The microprocessor programmes are selected by coded test cards. The twenty-place disposable cuvette disc is

**Evaluation de l'analyseur centrifugal ENI Gemeni contrôlé par microprocesseur.**

L'Electronucleonics Inc. Gemeni est un analyseur centrifugal contrôlé par microprocesseur de prix bas. Les programs du microprocesseur sont choisis à l'aide de cartes de test codées. Le disque de cuvetes à vingt places disponsible est rempli manue-

**Beurteilung des Eni Gemeni, ein Mikroprozessor-gesteuerter** Zentrifugal-Analysator

Der Elektronukleionik Inc Gemini ist ein billiger Zentrifugal-Analysator, der durch einen Mikroprozessor gesteuert wird. Die Mikroprozessor-Programme werden durch codierte Testkarten angewählt. Der wegwerfbare Küvetten-Halter mit 20 Plätzen manually loaded with the aid of a mechanical work station and pipettes. The system proved to be extremely simple to operate and, with the exception of the imprecision data for the estimation of calcium, the kits recommended for the system produced results of high quality.

llement à l'aide de pipettes et d'une station de travail mechanique. Le système est très simple à manier et à l'exception des résultats imprecis pour le calcium, les réactifs recommandés pour le système donnent des résultats de haute qualité.

wird manuell beschickt mit Hilfe einer mechanischen Arbeitsstation und Pipetten. Das System zeigte sich als sehr leicht bedienbar und die Reagenziensatze, die fur das System empfohlen werden, produzierten mit Ausnahme der Ungenauigkeiten bei der Bestimmung Calcium Resultate hoher Qualitat.

Page 206

**A microcomputer automated recording spectropolarimeter.** Victor C. Zadnik, James L. Scott, Robert Megargle, Julius Kerkay and Karl H. Pearson.

A Perkin-Elmer Model 241 polarimeter has been modified to accept a Bausch & Lomb double grating monochromator and a Bausch & Lomb 150 watt xenon light source. Modifications have been made to the monochromator and the polarimeter to assemble a unique computer automated scanning spectropolarimeter. The computer system used to interface to the spectropolarimeter was a MITS ALTAIR 8800A microprocessor with 48K byte of memory. Peripheral input/output devices include two MITS Model 88-DCD floppy disk drives, a Texas Instruments KSR-733 Silent 700 Teleprinter, and a Houston Instruments HI-PLOT digital plotter. Software written using MITS Disk Extended BASIC and appropriate assembly language subroutines allow for baseline corrections, repetitive scanning and differential or derivative ORD spectra.

**Spectropolarimètre automatisé, enregistrant avec microordinateur.**

Un polarimètre Perkin-Elmer 241 a été modifié de facon à être combiné avec un monochromateur à double réseau Bausch & Lomb et une lampe de xenon à 150 watt de Bausch & Lomb. Les modifications du polarimètre et du monochromateur permettent l'assemblage d'un unique spectropolarimètre automatique enregistrant. Le système d'ordinateur utilisé pour le couplage est un microprocesseur MITS ALTAIR 8800A avec une mémoire de 48K byte. La périphérie d'entrée et de sortie comprend deux unités pour floppy disk MITS 88-DCD, une imprimante Texas Instruments KSR-733 Silent 700 et un plotter digitale Houston Instruments, HI-PLOT. Le software écrit en MITS disk extended BASIC, qui utilise des sousprogrammes écrits en assembler, permet des corrections de ligne de base, multiples enregistrements, des spectres ORD différentiels ou derivés.

**Registrierendes, automatisiertes Spectropolarimeter mit Mikrocomputer**

Ein Perkin-Elmer Modell 241 Polarimeter wurde so modifiziert, dass es mit einem Bausch & Lomb Doppelgitter-Monochromator und einer Bausch & Lomb 150 Watt xenon Lampe kombiniert werden kann. Die Modifikationen am Monochromator und am Polarimeter erlaubten die Zusammenstellung eines einzigartigen computergesteuerten, registrierenden Spektropolarimeters. Der für die Kopplung mit dem Spektropolarimeter verwendete Mikrorechner ist ein MITS ALTAIR 8800A Mikroprozessor mit einem 48K byte Speicher. Die Ein/Ausgangs Peripherie umfasst zwei MITS Modell 88-DCD Floppy Disk Speichereinheiten, einen Texas Instruments KSR-733 Silent 700 Teleprinter, und einen Houston Instruments HI-PLOT Digital-Plotter. Die mit dem MITS Disk Extended BASIC geschriebene Software, die geeignete, in Assembler-Sprache geschriebene Unter-programme verwendet, erlaubt Basislinien-Korrekturen, mehrfaches Aufnegmen, Differenz-Spektren oder Aufzeichnung der ersten Ableitung von ORD–Spektren.

Page 214

**Principles of design, supply and usage of clinical laboratory equipment for primary health care in developing countries.** M. Hjelm, S.S. Brown and F.L. Mitchell.

This paper was prepared on the basis of a World Health Organisation meeting held in Geneva, Switzerland on 10-11 July 1978. The need for simple equipment suitable for primary health care is now realised and attempts are being made to develop instruments to cover the specific problems involved. The WHO sponsored a pilot project at the Clinical Research Centre, Harrow, UK, which aimed for the production of inexpensive and accurate photometers. The project was successful and the possibility of introducing the concept into developing countries was discussed.

**Les principes du dessein, de la fourniture et de l'usage de l'équipement du laboratoire clinique pour les soins de la santé primaires dans les pays développants.**

Cet article a été fondé sur la base d'une réunion de l'organisation de santé mondiale qui a eu lieu le 10-11 juillet 1978 en Genève dans la Suisse. On s'est rendu compte du besoin d'équipment simple adapté aux soins de santé primaires et on tâche de développer des instruments pour couvrir les problèmes spécifiques qui son entraînés. La OSM prend en charge un expérience au Clinical Research Centre, Harrow, Angleterre, dont le but est la production de photomètres précis et de bon marché. L'expérience a reussi et on a discuté la possibilité de présenter le concept dans les pays en développement.

**Bestandteile der Konstruktion, Lieferung, und Verwendung klinischer Laboratoriumsausrustung furPrimargesundheitsbehandlung in den Entwicklungsländern.**

Dieser Vortrag wurde auf der Basis einer Versammlung der Weltgesundheitsorganisation vorbereitet, die am 10-11 Juli 1978 in Geneve, Schweiz stattgefunden hat. Das Bedürfnis nach basisicher Ausrüstung geeignet für Primargesundheitsbehandlung ist jetzt bekannt geworden, und man versucht, Apparate zu entwickeln, umdie spezifischen Problemen zu lösen. Die Weltgesundheitsorganisation hat einen Versuchsplan an dem klinischen Forschungszentrum in Harrow, Grossbritannien finanziert, der die Produktion preiswerter und genauer Belichtungsmesser zu entwickeln versucht. Der Plan war erfolgreich, und man hat die Möglichkeit diskutiert, diesestionzept in den Entwicklungländern einzuführen.

Page 216

**A total systems approach to laboratory automation.** P.B. Stockwell.

The total systems approach is defined and then discussed with direct reference to the Laboratory of the Government Chemist's tar and nicotine levels in cigarette brands. A detailed specification for the complete analytical process including sampling pretreatment, measurement data processing and final reporting is prepared. The requirements of the analyst, the laboratory manager and the ultimate customer for the analytical results are integrated into the specification. The automation developed for the tar and nicotine survey work is also discussed.

**Une évaluation systématique et complète pour automation de laboratoire.**

Une évaluation systématique et complète est d'abord définie et ensuite discuté en connection avec la détermination du contenu en goudron et en nicotine de cigarettes de différentes marques effectues dans le Laboratory of the Government Chemist. Des spécifications détaillées sont déduites pour le processus analytique complet, y compris la préparation d'échantillons, le traitement des données et le protocole final. Les exigences du chimiste analytique, du chef de laboratoire et du client aux résultats analytiques sont intégrées dans les spécification. L'automation développée pour la détermination du contenu en goudron et en nicotine est également décrite.

**Ein umfassender System-Ansatz zur Laborautomation**

Ein umfassender System-Ansatz wird zuerst definiert und dann im direkten Zusammenhang mit Bestimmungen von Teer und Nikotin-Gehalt in Zigaretten verschiedener Marken, die im Laboratory of the Government Chemist durchgeführt werden, diskutiert. Detaillierte Spezifikationen für den vollständigen analytischen Prozess einschliesslich der Probenvorbereitung, der Messdatenverarbeitung und des Schlussprotokolls werden hergeleitet. Die Anforderungen seitens des Analytikers, des Laborleiters und des Endkunden an die analytischen Resultate sind in den Spezifikationen integriert. Die für die Durchführung der Beobachtung des Teer– und Nikotingehalts entwickelte Automation wird ebenfalls besprochen.

